# The direct and indirect effects of clinical empathy on well-being among pre-medical students: a structural equation model approach

**DOI:** 10.1186/s12909-021-02838-x

**Published:** 2021-08-02

**Authors:** Kelly Rhea MacArthur, Clare L. Stacey, Sarah Harvey, Jonathan Markle

**Affiliations:** 1 Department of Sociology and Anthropology, University of Nebraska Omaha, 6001 Dodge St., Omaha, NE 68182 USA; 2 Department of Sociology, Kent State University, 800 E. Summit St., Kent, OH 44242 USA; 3 Northeast Ohio Medical University, College of Medicine, 4209 St, OH-44, Rootstown, OH 44272 USA

**Keywords:** Empathy, Burnout, Depression, Pre-medical education, Medical socialization

## Abstract

**Background:**

Empathy is a well-established facet of clinical competency that research suggests is associated with enhanced medical student well-being. Since little is known about empathy and well-being *before* students enter medical school—during pre-medical education—the main goal of this study is to test a conceptual model of how clinical empathy is related to two indicators of well-being, depression, and burnout among pre-medical students. The theoretical model hypothesizes that three dimensions of clinical empathy—*Perspective-Taking, Compassionate Care,* and *Standing in Patients’ Shoes*— will be directly and negatively related to depression, as well as indirectly through its inverse relationship with three facets of burnout, *Emotional Exhaustion, Poor Academic Efficacy,* and *Cynicism.*

**Methods:**

Using survey data from a sample of 132 pre-medical students at an American Midwestern university, this study employs structural equation modeling (SEM) to test the theoretical model of the relationships between empathy, burnout, and depression among pre-medical students. We identify the direct effects of the three dimensions of the Jefferson Scale of Physician Empathy (JSE-S) on depression (CES-D), as well as the indirect effects of clinical empathy on depression through the three dimensions of the Maslach Burnout Inventory (MBI-S).

**Results:**

SEM analyses show that while none of the three dimensions of the JSE-S are directly related to depression, clinical empathy does significantly affect depression indirectly through burnout. Specifically, as predicted, we find that Perspective-Taking decreases Emotional Exhaustion, but, contrary to expectations, Compassionate Care increases it. And, the positive relationship between Compassionate Care and Emotional Exhaustion is particularly strong. In turn, Perspective-Taking and Compassionate Care are associated with depression in opposite directions and to different degrees.

**Conclusions:**

Findings suggest that clinical empathy as measured by the JSE-S produces both positive and negative effects on personal well-being. We conclude that further conceptual clarity of clinical empathy is needed to better discern how the different dimensions impact different indicators of well-being. Given that pre-medical education is a crucial time for emotional socialization, the challenge for medical education will be fostering the positive, cognitive aspects of clinical empathy while simultaneously mitigating the adverse effects of affective empathy on medical student well-being.

## Background

Empathy, generally defined as the capacity to understand the perspectives and feelings of others, is a well-established facet of clinical competency and quality doctor-patient relationships. Research shows that a physician’s ability to communicate empathy is related to increased patient satisfaction [1] improved clinical outcomes [1], increased patient enablement [2], decreased patient distress [3], and lower burnout and depression among physicians [3]. Despite its importance for both patient and physician well-being, a number of studies identify levels of empathy that may begin relatively high upon entry into medical school, but then subsequently decline over time throughout medical training [4, 5], alongside measures of personal well-being that also seem to worsen [6]. Little is known about empathy and well-being *before* students enter medical school— during pre-medical education—which is a crucial time of emotional socialization for aspiring physicians and, as Cundell notes, represents an important opportunity for early cognitive empathy training [7]. This study, therefore, constitutes a vital first step in assessing empathy in this largely understudied population, as it examines how different aspects of clinical empathy relate to measures of well-being among pre-medical students.

### Measuring clinical empathy

In response to the need to measure empathy in clinical settings, Hojat and colleagues developed the Jefferson Scale of Physician Empathy (JSE) [8]. They subsequently adapted the original JSE for physicians (HP-Version), medical students (S-Version), and other health professions students (HPS-Version). The JSE-S Version captures three underlying components of clinical empathy, that of Perspective-Taking (viewing a situation from another’s point of view), Compassionate Care (emotions in patient care), and Standing in Patients’ Shoes (thinking like a patient). Clinical empathy in the JSE is conceptualized as cognitive rather than affective, meaning that physicians understand patients’ experiences and communicate that understanding without experiencing the emotional state themselves [9].

The JSE-S has been extensively tested for its reliability and validity among medical students [8]. Numerous exploratory factor analyses (EFAs) confirm the three-factor latent structure of the JSE-S as it was originally theorized, as do the few existing confirmatory factor analyses (CFAs) [10–18]. A small number of studies, however, identify two [19], four [20], and five factors [21]. Even in studies that identify three latent factors in the JSE-S, only a few analyze the dimensions separately for their relationships with other variables. In an exhaustive literature search, we identified seven studies that examine the three factors separately among medical students. They find different relationships across the three dimensions with respect to gender [16], rates of change over time [20], other measures of empathy [22], willingness to show empathetic behavior [17], and burnout [23]. Several studies also show that only certain aspects of empathy decline, while others may actually improve [20]. Given that student empathy varies depending on the stage of training, it is important to develop a measurement strategy that examines the three “factors independently in a context dependent way” [20]. One such context is the pre-medical experience, for which research has not established baseline levels of empathy. To fully understand the phenomenon of clinical empathy in medical trainees, we argue that greater attention should be paid to empathy in pre-medical students, as well as to the ways that the three dimensions of clinical empathy in the JSE-S may differentially impact well-being outcomes for students.

### Medical student well-being

Burnout and depression are prevalent among medical students [24–26], even among first-year students [27]. The majority of studies show that empathy is associated with less burnout and depression [3, 28–30]^,^ suggesting that relationships with patients can serve as a buffer to the stresses of medical training [31]. Nonetheless, some studies do not report an association [32], and there are concerns that the emotional labor associated with clinical empathy can produce compassion fatigue/exposure to vicarious trauma [33]. While a few researchers have measured the well-being of pre-medical students, no studies have explored the link between levels of empathy and burnout/depression for these students. One study, however, shows that a mindfulness intervention improved both depression and empathy among pre-medical students [34].

Research on pre-medical students is limited and tends to focus on reasons for attrition and stereotypes of the pre-medical personality [35]. Two existing studies indicate that pre-medical students have higher levels of burnout and depressive symptoms compared to their non-pre-medical counterparts [36, 37]. In a non-comparative study among pre-medical students, Grace finds that burnout and depression are negatively associated with interest in medical school [38]. There is also evidence that those with lower baseline empathy upon entrance into medical school experience greater decline in empathy thereafter, although this is inconclusive [4], suggesting, as some other studies have, that physicians’ poor health and less than ideal levels of clinical empathy likely begin before students enter medical school [36–39].

**Fig. 1:**
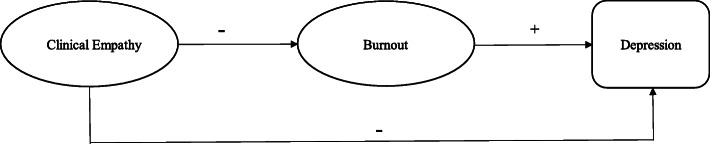
Conceptual Model of the Hypothesized Relationships between Clinical Empathy and Well-Being

Establishing baseline levels of clinical empathy and examining the relationship between empathy and well-being during the pre-medical period is important in order to develop appropriately timed interventions. Thus, this study aims to 1) identify baseline levels of clinical empathy, burnout, and depression among pre-medical students; and 2) examine how different aspects of clinical empathy are related to well-being. Specifically, based on the literature discussed above, and is depicted in the conceptual model in Fig. 1, we hypothesize that the three empathy dimensions will have both direct relationships with depression, as well as indirect relationships through three facets of burnout. The results of this study will help establish baseline levels of clinical empathy and well-being among pre-medical students and contribute to ongoing debates about the existence and extent of empathy declines during medical training [2, 40–43].

## Method

### Participants and procedure

Survey data were collected in April of 2019 at a large state Midwestern university in the United States as part of a larger longitudinal, mixed-methods study on clinical empathy development and decline. Participants were recruited among all undergraduate students who were enrolled in the four-year pre-medical program that provides a clear curricular pathway to students interested in applying to medical school. Online invitations to the confidential and anonymous Qualtrics survey were sent to 666 undergraduate students, 45 of whom received a slightly modified version of the survey as to make a few of the questions applicable to the early admittance program in which they were enrolled. A total of 132 students (28 BS/MD students) consented to participate, constituting an approximate 20% response rate. There were no missing data on any of the variables used in these analyses except for one individual who identified their gender as “queer” and so we substituted the mean (male) for that individual. Informed consent was obtained for all participants and all study procedures were approved by the Institutional Review Board (#18–503).

### Measures

#### Clinical empathy

The JSE-S Version contains 20 items on a 7-point Likert scale in which respondents are asked to rate their agreement (1 = strongly disagree to 7 = strongly agree). The medical student version words items in third person (i.e., refers to “physicians” rather than “I”). Half of the items are positively worded and half of them are negatively worded as to reduce passive response [13]. Items were recoded so that all items indicate higher degrees of clinical empathy.

#### Well-being

We operationalize well-being as burnout and depressive symptoms. The main well-being dependent variable in this study is depression, measured with the Center for Epidemiologic Studies Depression scale (CES-D), which is a summative scale ranging from 10 to 40 that asks respondents how often they have experienced depressive symptoms in the past week (1 = Most or all of the time to 4 = Rarely or none of the time) [44]. Some items were reverse-coded so that higher values reflect more depressive symptoms. The CES-D has been used among pre-medical students with high internal consistency [38].

We use burnout as a second indicator of well-being, using the 15-item Maslach Burnout Inventory-Student Survey (MBI-SS) that includes three dimensions of burnout: Emotional Exhaustion, Academic Efficacy, and Cynicism [45]. The items ask respondents to rate how frequently they may have had certain feelings about their academic work (0 = never had this feeling to 6 = every day), with the total summed scale ranging from 0 to 84. Summed sub-scales for the three dimensions range from 0 to 30 for the Emotional Exhaustion scale; 0–36 for the Poor Academic Efficacy scale; and 0 to 18 for the Cynicism scale. The three subscales have been validated among pre-clinical medical students [46] and the two studies that employ the MBI-SS among pre-medical students show high internal consistency [37, 38].

### Data analysis

To confirm the 3-latent structure of the JSE-S among pre-medical students, we conducted a CFA in Structural Equation Modeling (SEM) because it can simultaneously examine multiple dependent variables, model latent variables, and estimate indirect effects [47]. We first ran three nested models with all 20 items and then examined the 20-item global latent variable of clinical empathy in comparison to 2- and 3- factor structures. [analyses available upon request]. After confirming the 3-factor latent structure of the JSE-S among this sample of pre-medical students, we then tested direct and indirect pathways from empathy to well-being. As shown in Fig. 1, we hypothesize that clinical empathy will be directly and negatively related to depression, as well as negatively associated with depression through its inverse relationships with burnout. We control for women’s greater levels of depression compared to men [48]. For all models, we estimate the covariance between the latent variables of the JSE-S and the MBI-S; use Maximum Likelihood Estimation (MLE); and set one of the items to 1 as to scale the latent variable.

For all of the SEM models, we report several fit indices, including the Chi-Square Statistic (χ^2^), the Comparative Fit Index (CFI), the Root Mean Squared Error of Approximation (RMSEA), and the Standardized Root Mean Squared Residual (SRMR). A statistically significant χ^2^ is undesirable, as it indicates that the hypothesized model is significantly different than the observed data. Higher values of the CFI (>.95) indicate better model fit, while lower values of RMSEA (<.06) and SRMR (<.08) suggest better fit. For all analyses, we set our alpha at .05. Descriptive statistics were computed using IBM SPSS Statistics 26 and SEM was conducted using Muthén & Muthén Mplus 7.31.

## Results

As shown in Table 1, the sample of 132 pre-medical students is predominantly female (67%), white (72%), and not married (97%). The average age is about 20 years old and they tend to have parents with advanced degrees (33% of their mothers and 45% of their fathers). The students in the sample range from being freshman (27%) to seniors (14%) and about 70% reported having a GPA of 3.5 or higher on a 4.0 scale.

**Table 1:** Descriptive Statistics for Clinical Empathy and Well-Being (*N* = 132)

Construct	Properties	Mean (SD)	95% C.I.
Clinical Empathy (JSE-S Version)	20 items, *a* = .80, range: 20–140, 1 = Strongly disagree to 7 = Strongly agree	5.52 (.58)	5.42–5.62
Perspective-Taking	6 items, *a* = .74, range: 6–42, 1 = Strongly disagree to 7 = Strongly agree	5.84 (.72)	5.72–6.97
Compassionate Care	4 items, *a* = .64, range: 4–28, 1 = Strongly disagree to 7 = Strongly agree	5.89 (.86)	5.75–6.04
Standing in Patients’ Shoes	2 items, *a* = .74, range: 2–14, 1 = Strongly disagree to 7 = Strongly agree	4.70 (1.25)	4.49–4.92
Depression (CES-D-10)	10 items, *a* = .87, range:10–40, 1 = rarely or none of the time to 4 = most or all of the time	1.93 (.64)	1.82–2.05
Burnout (MBI-SS)	14 items, *a* = .88, range: 0–84, 0 = strongly disagree to 6 = strongly agree	2.18 (1.08)	1.10–2.37
Emotional Exhaustion	5 items, *a* = .92, 0–30, 0 = strongly disagree to 6 = strongly agree	3.27 (1.59)	2.10–3.55
Poor Academic Efficacy^a^	6 items, *a* = .83, range: 0–36, 0 = strongly disagree to6 = strongly agree	1.56 (1.12)	1.37–1.76
Cynicism	3 items, *a* = .86, range: 0–18, 0 = strongly disagree to 6 = strongly agree	1.92 (1.72)	1.63–2.22

^a^The Academic Efficacy sub-scale was re-named “Poor Academic Efficacy” to reflect that some items were reverse-coded and so higher values reflect more burnout

### Well-being descriptive statistics

Pre-medical students reported a mean of 1.93 (sd: 64) on the summed and averaged CES-D scale that ranges from 1 to 4, which is the equivalent of a mean of 19.35 (sd: 6.43) not averaged that ranges from 10 to 40. Students reported an average of 2.18 (sd: 1.08) on the MBI-SS on a scale of 0 to 6. Of the three dimensions of burnout, students reported the highest levels of Emotional Exhaustion (mean: 3.27, sd: 1.59) and the lowest levels of Poor Academic Efficacy (mean: 1.56, sd: 1.12). In examining the 95% confidence intervals, students reported significantly more Emotional Exhaustion than Cynicism, but not Poor Academic Efficacy.

### Measurement model

After confirming the latent 3-factor structure of the JSE-S among pre-medical students, we then estimated the entire measurement model with both the JSE-S and the MBI-SS. Because of the relatively small sample size compared to the number of variables, we used the CES-D 10-item scale as an observed, rather than latent, variable in the SEM analyses, which is reasonable given that this scale is a widely used and extensively validated instrument to measure depressive symptoms in the general population and has been implemented on medical students and pre-medical students. In addition to the high internal consistency of the scale (*a* = .87), we confirmed that the scale was valid in this sample using CFA in SEM. Standardized factor loadings for the CES-D ranged from .50 to .81 and, with one added correlation between Items 5 and 8, all model fit indices were acceptable (χ^2^ = 48.48, df = 34, CFI: .97, RMSEA: .06, SRMR: .04) [analyses not shown].

**Fig. 2:**
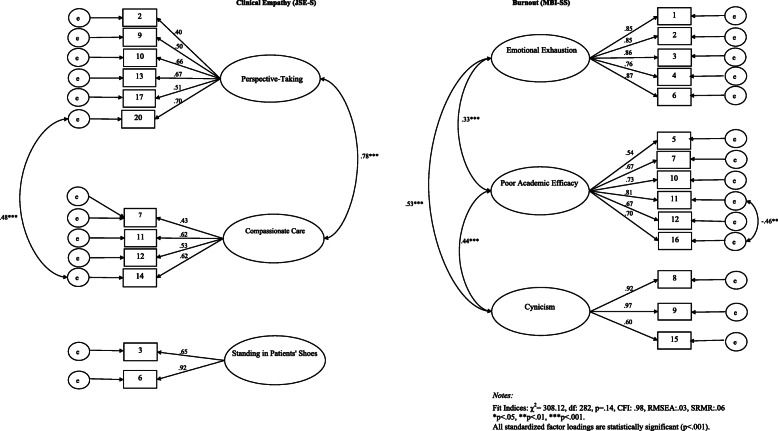
Measurement Model: CFA Standardized Factor Loadings

When we ran the initial measurement model, the statistically significant chi square model fit statistic indicated that the measurement model was not an acceptable fit to the data (χ^2^ = 505.73, df = 389, CFI: .93, RMSEA: .05, SRMR: .06). To solve this issue of poor model fit, we took the suggestion of the modification indices to add a correlation between items MBI11 & MBI16. To further improve model fit, and in the interest of parsimony, we then trimmed items from the model that had low factor loadings, as other studies using the JSE have done (see Table 1 for which items were cut/retained) [12, 19]. The final measurement model is depicted in Fig. 2; it had good fit indices (χ^2^ = 308.12, df = 282, CFI: .98, RMSEA: .03, SRMR: .06) and contained items with standardized factor loadings that were all >.40 and statistically significant (*p* < .001).

### Structural model

**Fig. 3:**
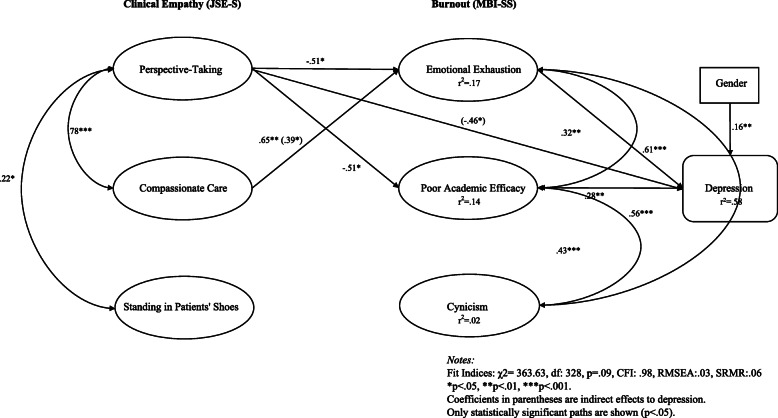
Standardized Coefficients for Direct and Indirect Effects of Clinical Empathy on Well-Being

We next tested the structural model. This model explained 58.2% of the variation in depression among pre-medical students and the relationships between empathy, burnout, and depression generally functioned in the hypothesized manner depicted in Fig. 1, as indicated by the good model fit indices (χ^2^ = 363.63, df = 328, CFI: .98, RMSEA: .03, SRMR: .06). As shown in Fig. 3, all three of the latent factors of the MBI-SS were positively and significantly related to each other, but between the three latent factors of the JSE-S, Standing in Patients’ Shoes was not significantly correlated with Compassionate Care.

#### Direct effects

Figure 3 reports the standardized regression weights and shows that, contrary to hypotheses (see Fig. 1), we did not find that any of the three types of empathy had direct relationships with depression (*p* > .05). However, as predicted, we found that, controlling for women’s greater degree of depressive symptoms (β = .16, *p* = .01), burnout had a positive relationship with depression in that there was a strong relationship between Emotional Exhaustion and depression (β = .61, *p* < .000). Poor Academic Efficacy was also positively associated with depression, but not as strongly (β = .28, *p* = .00), and Cynicism was not significantly related to depression (β = .03, *p* = .76).

#### Indirect effects

As shown in Fig. 3, empathy and burnout were significantly related. As predicted, greater Perspective-Taking was negatively associated with Poor Academic Efficacy (β = − 51, *p* = .02) and Emotional Exhaustion (β = −.51, *p* = .05). Compassionate Care was also strongly related to burnout, but in a direction counter to that was hypothesized (see Fig. 1) in that the more pre-medical students value Compassionate Care, the more likely they were to be emotionally exhausted (β = .65, *p* = .01). Standing in Patients’ Shoes was not significantly related to any of the three dimensions of burnout (*p* > .05).

**Fig. 4:**
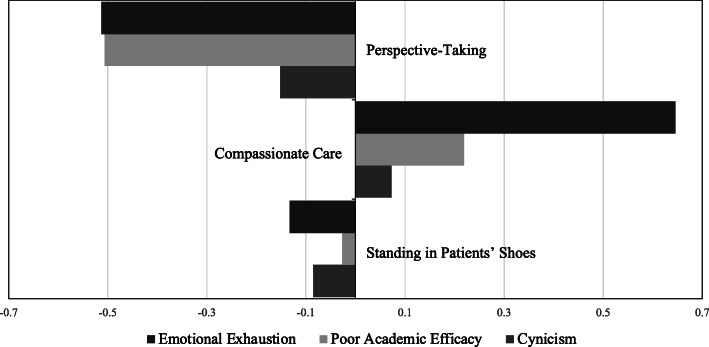
Relative Effect Sizes (β) of Relationships between Clinical Empathy & Burnout

In assessing the relative strength of relationships between the three dimensions of empathy and the three types of burnout, Fig. 4 illustrates that Emotional Exhaustion had the strongest relationship with Compassionate Care (β = .65), followed by Perspective-Taking (β = −.51), neither of which had strong relationships with Cynicism (β = .07, β = −.15). Standing in Patients’ Shoes had weak relationships with all measures of burnout (β = −.13, β = −.03, β = −.09).

While we did not identify any direct effects between clinical empathy and depression, there were several significant indirect pathways from Perspective-Taking and Compassionate Care, but not Standing in Patients’ Shoes. Perspective-taking had an indirect, negative association with depression (total indirect effects: β = −.46, *p* = .03). Compassionate care also had an indirect, positive relationship with depression (total indirect effects: β = .46, *p* = .03), mostly through its relationship with Emotional Exhaustion (β = .39, *p* = .03). Thus, while those who value Perspective-Taking experienced less depression, those who value Compassionate Care experienced more depression because of their greater Emotional Exhaustion.

## Discussion

All in the same year, Hojat and LaNoue advocated for more studies “in different sociocultural environments, populations, and in different translated versions of the scale to assure the psychometric soundness of the JSE in a variety of situations” [14], Leombruni and colleagues called for the examination of the subcomponents of empathy separately [15]; and Paro and colleagues noted the dearth of studies establishing a link between empathy and burnout [30]. Since the publication of these studies in 2014, very few have taken up these charges. We, however, did so in the American pre-medical context by, first, identifying baseline levels of clinical empathy, burnout, and depression among pre-medical students and, second, by examining how different aspects of clinical empathy are related to well-being.

### Establishing baseline levels of clinical empathy and well-being

Pre-medical students in this sample report a mean of 110 for the global 20-item JSE-S, which is slightly lower than average clinical empathy scores of first year medical students in the U.S. (that range between 114 and 115 with standard deviations from 9 to 12) [8]. It is possible that pre-medical students have lower levels of clinical empathy than medical students or that pre-medical students with higher clinical empathy are more likely to be accepted to, and actually attend, medical school. Future research should compare pre-medical students to medical students to determine to what degree a difference exists.

With respect to well-being, we find that levels of depression among pre-medical students are slightly higher than levels reported in the only other study that uses the CES-D in this population [38]. We find slightly lower means in the Emotional Exhaustion, Poor Academic Efficacy, and Cynicism burnout sub-scales than Fang and colleagues [37], whereas our data are consistent with previous studies that show pre-medical students score highest in levels of Emotional Exhaustion and lowest in Cynicism [26, 37]. Given that those who are at higher risk for burnout and depression might be more likely to be on the pre-medical track than undergraduates who are not [36, 37] baseline levels found here could help future research to distinguish between selection effects into medical school and the effects of the structure and culture of medical education on the changes in clinical empathy and well-being throughout medical training.

### Clinical empathy: good or bad for well-being?

SEM analyses show that over half of the variation in depression among pre-medical students is explained by clinical empathy and burnout, with empathy affecting depression primarily through burnout as opposed to directly. Although it was counter to our hypotheses concerning direct effects (see Fig. 1), this finding is not surprising given the evidence showing the factors that contribute the most to depression among medical students are those that are related to academic stressors [49].

The existence, direction, and strength of relationships are not the same between the three factors of the JSE-S and the three types of the MBI-SS. We find that the more students take the perspective of others, the less emotional exhaustion they experience, which in turn lessens depression. By contrast, students who report more Compassionate Care have greater Emotional Exhaustion, which then leads to greater depression. Thus, while being able to take patients’ perspectives is beneficial for burnout and depression among pre-medical students, Compassionate Care has the opposite effect—and this positive relationship between Compassionate Care and Emotional Exhaustion is particularly strong.

It is difficult to discern precisely why Compassionate Care produces deleterious outcomes relative to Perspective-Taking, which is contrary to our hypotheses about the positive relationship between empathy and well-being (see Fig. 1). Nonetheless, a study by Ünlü and Uludağ may provide some insight that supports our counterintuitive finding regarding the negative effects of clinical empathy [50]. Using the JSE-S among a sample of medical students, they find that Perspective-Taking and Compassionate Care were significantly and positively correlated with avoiding and having difficulty communicating about death/dying among patients [50]. We concur with Ünlü and Uludağ that these findings reflect a lack of conceptual distinction between cognitive and affective empathy in the JSE-S [50]. In fact, in his book, Hojat and colleagues define Compassionate Care as having two components, understanding patient experiences and emotions in patient care, suggesting that the factor is comprised of both cognitive and affective dimensions of empathy [8]. Indeed, three of the items associated with Compassionate Care appear to measure what Davis labels empathic concern, or the tendency to experience the feelings of others or feel sympathy/compassion for unfortunate people [51]. As such, our finding that more Compassionate Care increases Emotional Exhaustion, and in turn depression, corroborates previous research that links affective empathy to compassion fatigue, burnout, and vicarious trauma [8].

Results suggesting that greater empathy leads to compassion fatigue also prompt questions about previous studies that report a positive relationship between empathy and well-being [3, 28, 29, 52]. Rather than reflecting the negative effects of too much empathy, the use of the global JSE-S scale in past studies may in fact obscure the existence and strength of an inverse relationship between certain aspects of empathy and burnout. This argument is consistent with Hojat and LaNoue’s conclusion that “because of its cognitive nature, abundance of empathy is always beneficial in patient-physician relationships; understanding in excess cannot be detrimental” [13]. In other words, findings here should not be interpreted as actual negative effects of clinical empathy, but rather an issue of methodological operationalization. As other scholars have suggested [15, 20], the current study provides further support for the claim that the different dimensions of clinical empathy should be examined separately and, in particular, the affective and cognitive aspects, which do not seem to develop and/or decline uniformly [53, 54]. Conceptual clarity is needed with respect to the three different components of clinical empathy as measured by the JSE-S so that researchers can better discern how different dimensions of the cognitive and affective aspects of empathy impact medical student well-being. Qualitative analyses may compliment studies using the JSE-S in deciphering the effects of the different aspects of clinical empathy on well-being [55, 56].

### Future research

This study has several limitations that future research should consider. First, it is likely that some proportion of our sample will never attend medical school. Pre-medical students who leave the medical track may be different from their peers who enter medical school, raising questions about the utility of our data when comparing pre-meds to medical students. A small number of respondents in the sample are also early admission students who may have a qualitatively different experience in medical school and therefore differ from their traditional pre-med peers with respect to empathy and/or well-being. Studies like the current one, however, can contribute to understandings of the reasons for a loss of interest in pursing a career in medicine [57].

Our study is also limited by a small sample that required us to correlate a few items in order to achieve adequate model fit, although several other studies using the JSE have proceeded similarly [11, 13, 18]. Additionally, the latent factor of Standing in Patients’ Shoes does not correlate with the other two JSE-S latent factors in the measurement model or with Compassionate Care in the structural model, suggesting that there may be other latent factors in addition to the three identified here. Finally, as with all studies that use the JSE-S, this study relies on self-reports of empathy and therefore is subject to social desirability bias. Self-reports can be crude measures of student/physician behavior in the context of clinical encounters, but there is evidence that scores on the JSE-S are related to clinical competence rated by an observer [58], as well as to patient perceptions of physician self-reported empathy [59].

Since the current study is cross-sectional, it does not allow for strong causal claims or the examination of the enduring effects of clinical empathy scores on behavior. Although SEM implies causal pathways, it is important to note that longitudinal data is necessary to establish causal order. It is possible, and even likely, that burnout and depression affect clinical empathy instead of the direction that it is modeled here, as some studies have shown [23, 29, 60]. Given these limitations, future research should replicate these analyses on larger samples of pre-medical students. Furthermore, given recent evidence that personality factors affect the *trajectory* of empathy throughout medical school [61], future research should examine the relationships between empathy and well-being longitudinally and while controlling for a larger range of factors, most notably personality factors [22, 62].

Despite its limitations, this study is one of very few studies to examine the differential effects of the three dimensions of JSE-S on well-being outcomes [22, 23]. This is also one of a few studies of clinical empathy among students [12, 13, 16] to use SEM, which is an underutilized, but potentially valuable tool, in medical education research [63]. Given the lack of conceptual clarity between burnout and depression [25], SEM is an especially superior method to other linear models that can neither simultaneously examine the effects of clinical empathy on multiple measures of well-being nor estimate indirect effects on outcomes. Lastly, this was the first study to employ the JSE-S on pre-medical students, which arguably opens the door for the inclusion of pre-medical students in subsequent studies about clinical empathy development. Since medical students do not matriculate as “blank slates,” it is imperative that we consider empathy development prior to the M1 year.

## Conclusions

The findings of this study have several implications for protecting the well-being of future physicians. Our finding that the effect of clinical empathy on depression is solely indirect warrants a continued focus on burnout in pre-medical and medical education. Nonetheless, the indirect effects of clinical empathy on depression through burnout identified here suggest a need to upstream to the antecedents of burnout. Focusing on clinical empathy development might be one effective way to protect against burnout and depression, while also improving future patient care [1, 2, 64]. In addition to enhancing personal well-being, another motivation for pre-medical education to focus on empathy development is that research shows a connection between a variety of emotions—e.g., hope, pride, anxiety, and shame—and academic achievement in medical school [65].

Given that greater compassionate care appears linked to greater emotional exhaustion and depression, it behooves medical educators to emphasize the cognitive aspects of empathy (i.e., perspective-taking) that our findings suggest protect against the adverse effects of the demands of medical school on students’ well-being. For early medical students who often possess abstract and idealized notions of the doctor-patient relationship [31], it would be beneficial to provide them with opportunities to learn about affective versus cognitive empathy, especially since they themselves seem to distinguish between the emotional versus intellectual parts of empathy development [54]. As Harvey notes, pre-medical students are subjected to a hidden/informal curriculum that touts emotional readiness but does very little to prepare students for the emotional demands of medical school [66], which in part explains why some studies show that medical students crave more explicit discussion around emotions in medical school [67, 68] and that they would likely benefit from more emotional socialization in their pre-medical years [9, 69]. Cognitive empathy training seems especially important given a recent meta-analysis that finds empathy interventions in medical education are indeed effective [70].

In addition to this study having implications for the well-being of medical students and cognitive empathy training, findings presented here may be of use to medical school educators and administrators, who are increasingly interested in recruiting students with strong non-cognitive skills. As such, many medical schools now use situational judgement tests (SJTs) to assess student readiness for professional training and socialization [71]. But what of emotional readiness? Our data suggest that despite having little to no clinical training or experience, students in our sample have mean clinical empathy scores on par with medical students and residents and that those students scoring higher in Compassionate Care are more likely to experience Emotional Exhaustion, relative to those students engaging in Perspective-Taking. These findings suggest that pre-medical students are not empathic “blank slates” onto which schools impart understanding. Rather, students have a priori perceptions of empathy that form over-time, akin to what sociologists call “emotional capital” [66]. This capital continues to accrue throughout medical school but can also be undermined via curriculum, burnout, and other stressors. Recognizing empathy as an accrued resource may prompt medical school administrators to assess the clinical empathy of prospective or incoming students, perhaps using the JSE-S that is employed here. Data could then be shared with an incoming class to promote an institutional culture that encourages and supports a thoughtful approach to empathy training in medical students.

In conclusion, this study shows that students come to medical school with already-developing understandings of the doctor-patient relationship and that these understandings are linked to well-being in both positive and negative ways. As students make the transition from pre-medical to medical school, the challenge for medical education, then, will be how to train students to have the beneficial, positive aspects of clinical empathy without experiencing the adverse effects of affective empathy.

## Data Availability

The datasets generated and/or analyzed during the current study are not publicly available due to ongoing data analysis, but are available from the corresponding author on reasonable request.

## Abbreviations

JSE-S: Jefferson scale of clinical empathy student version
EFA: Exploratory factor analysis
CFA: Confirmatory factor analysis
CES-D: Center for epidemiologic studies depression scale
MBI-SS: Maslach burnout inventory-student survey
SEM: Structural equation modeling
MLE: Maximum likelihood estimation
RMSEA: Root mean squared error of approximation
CFI: Comparative fit index
SRMR: Standardized root mean squared residual
